# Cerebrospinal Fluid Cortisol Mediates Brain-Derived Neurotrophic Factor Relationships to Mortality after Severe TBI: A Prospective Cohort Study

**DOI:** 10.3389/fnmol.2017.00044

**Published:** 2017-03-09

**Authors:** Miranda J. Munoz, Raj G. Kumar, Byung-Mo Oh, Yvette P. Conley, Zhensheng Wang, Michelle D. Failla, Amy K. Wagner

**Affiliations:** ^1^Department of Physical Medicine and Rehabilitation, University of PittsburghPittsburgh, PA, USA; ^2^Department of Biological Sciences, Carnegie Mellon UniversityPittsburgh, PA, USA; ^3^Department of Epidemiology, University of PittsburghPittsburgh, PA, USA; ^4^Department of Rehabilitation Medicine, Seoul National University HospitalSeoul, South Korea; ^5^Department of Nursing, University of PittsburghPittsburgh, PA, USA; ^6^Safar Center for Resuscitation Research, University of PittsburghPittsburgh, PA, USA; ^7^Department of Psychiatry, Vanderbilt University Medical CenterNashville, TN, USA; ^8^Department of Neuroscience, University of PittsburghPittsburgh, PA, USA; ^9^Center for Neuroscience, University of PittsburghPittsburgh, PA, USA

**Keywords:** cortisol, BDNF, TBI, biomarkers, genetics, cerebrospinal fluid

## Abstract

Distinct regulatory signaling mechanisms exist between cortisol and brain derived neurotrophic factor (BDNF) that may influence secondary injury cascades associated with traumatic brain injury (TBI) and predict outcome. We investigated concurrent CSF BDNF and cortisol relationships in 117 patients sampled days 0–6 after severe TBI while accounting for BDNF genetics and age. We also determined associations between CSF BDNF and cortisol with 6-month mortality. *BDNF* variants, rs6265 and rs7124442, were used to create a gene risk score (GRS) in reference to previously published hypothesized risk for mortality in “younger patients” (<48 years) and hypothesized BDNF production/secretion capacity with these variants. Group based trajectory analysis (TRAJ) was used to create two cortisol groups (high and low trajectories). A Bayesian estimation approach informed the mediation models. Results show CSF BDNF predicted patient cortisol TRAJ group (*P* = 0.001). Also, GRS moderated BDNF associations with cortisol TRAJ group. Additionally, cortisol TRAJ predicted 6-month mortality (*P* = 0.001). In a mediation analysis, BDNF predicted mortality, with cortisol acting as the mediator (*P* = 0.011), yielding a mediation percentage of 29.92%. Mediation effects increased to 45.45% among younger patients. A BDNF^*^GRS interaction predicted mortality in younger patients (*P* = 0.004). Thus, we conclude 6-month mortality after severe TBI can be predicted through a mediation model with CSF cortisol and BDNF, suggesting a regulatory role for cortisol with BDNF's contribution to TBI pathophysiology and mortality, particularly among younger individuals with severe TBI. Based on the literature, cortisol modulated BDNF effects on mortality after TBI may be related to known hormone and neurotrophin relationships to neurological injury severity and autonomic nervous system imbalance.

## Introduction

Although death rates have decreased over time, ~2.5 million Americans experience traumatic brain injury (TBI) yearly, with more than 50,000 associated fatalities (Centers for Disease Control Prevention, 2016). TBI results in several acute secondary injury cascades, that include aseptic inflammation (Kumar et al., 2015, 2016), excitotoxicity (Wagner et al., 2005), monaminergic dysfunction (Wagner et al., 2007), neurotrophin abnormalities (Failla et al., 2016), and stress induced steroidogenesis (Wagner et al., 2011a; Santarsieri et al., 2014) that result in blood brain barrier disruption and CNS damage (Wagner et al., 2011b; Goyal et al., 2013); representative biomarkers for each of these pathways have been identified in CSF and serum for clinical populations with moderate/severe TBI. However, individual biomarker studies have limitations with predicting therapeutic treatment response (Maas et al., 2010), which may be due to the focus of current research on single biomarker relationships, rather than the interactions of several. Further, many clinical intervention studies have not included biomarker characterization to assess treatment effects.

Under certain conditions glucocorticoids, cortisol in humans and corticosterone in animals, can be neuroprotective, anti-inflammatory, and anticonvulsive, in order to restore homeostasis after injury, as the end product of the stress-activated HPA axis (McEwan, 1999; Jeanneteau et al., 2008; Joëls, 2008). Based on this premise, the Corticosteroid Randomization after Significant Head Injury (CRASH) trial administered the glucocorticoid methylprednisolone acutely after TBI. This trial was stopped prematurely when treatment had a higher mortality rate than placebo (Roberts et al., 2004). Recent work suggests elevated cortisol levels, when linked to prolonged stress, can impair synaptic plasticity and can result in neuronal cell death (Antonawich et al., 1999; Rothman and Mattson, 2013). Also, our previous work shows endogenous CSF cortisol levels are markedly increased after TBI, and we showed that sustained elevations in CSF cortisol profiles are associated with poorer outcomes (Santarsieri et al., 2014).

Brain derived neurotrophic factor (BDNF) is well-known for its roles in neuronal survival, neuronal maintenance, and neural plasticity. Despite these benefits, BDNF administration after experimental TBI was not protective against structural or functional deficits (Blaha et al., 2000). Subsequent work suggests that BDNF effects on TBI pathophysiology may be target receptor dependent, with the pro-apoptotic p75 receptor upregulation post-injury contributing to cell death (Rostami et al., 2014; Sebastiani et al., 2015). Our previous clinical work suggests CSF BDNF levels are elevated after TBI and associated with earlier time until death (Failla et al., 2016).

Brain derived neurotrophic factor (BDNF) and cortisol regulation are interconnected via multiple mechanisms (Rothman and Mattson, 2013), and levels of each are modified under stress, which are relevant in the context of TBI, a major pathophysiological stressor. While the exact pathways by which cortisol and BDNF interact are not completely known, some work shows glucocorticoid response elements are located in the *BDNF* gene promoter region, suggesting transcriptional control over BDNF production (Rothman and Mattson, 2013). Other indirect mechanisms propose glucocorticoid regulation of BDNF through CREB, MAPK/ERK, or Shp2 (Kumamaru et al., 2008, 2011; Alboni et al., 2011). BDNF, through synaptic modulation in the nucleus of the solitary tract (Clark et al., 2011) and hypothalamus (Tapia-Arancibia et al., 2004), may also regulate HPA-axis reactivity and cortisol releasing hormone (Jeanneteau et al., 2012). Interactive signaling between BDNF and cortisol may significantly influence HPA-reactivity immediately after TBI. The literature on regulatory effects between cortisol and BDNF is sparse, yet one experimental TBI study, involving young adult rats, does suggest that both injury and adrenalectomy independently resulted in acute (within 4 h of injury) elevations in hippocampal BDNF mRNA expression, and the effects of both TBI and adrenalectomy on BDNF elevations are cumulative. Alternatively, corticosterone replacement prevented this increase among adrenalectomized rats (Grundy et al., 2000). Together, these results suggest that cortisol can have acute modulatory effects that limit increased BDNF expression after TBI. Similar effects are noted with NT3 (Grundy et al., 2001).

Both cortisol and BDNF exhibit age-dependent changes that may impact their role with damage and recovery after TBI, especially as older age increases risk for mortality after TBI (Roozenbeek et al., 2012). Positive correlations between high cortisol levels and older age are likely due to age-associated HPA axis changes, including decreased cortisol clearance and impaired glucocorticoid negative feedback (Ferrari et al., 2001; Pal et al., 2014). Older age is also linked to lower BDNF levels (Erickson et al., 2012) and relative brain increases in the pro-apoptotic p75 receptor (Webster et al., 2006; Tapia-Arancibia et al., 2008). In populations with TBI, both cortisol and BDNF levels vary by age (Santarsieri et al., 2014; Failla et al., 2016), and our previous work characterizes interactions between *BDNF* gene variation and age that predict mortality post-TBI, wherein risk variants at a particular gene locus differ by age (Failla et al., 2016).

The, *BDNF* genotypes (Val/Val) for rs6265 causes an amino-acid substitution of valine to methionine at amino-acid residue 66 (Val66Met) (Tapia-Arancibia et al., 2004). This substitution alters intracellular trafficking and packaging of pro-BDNF and secretion of the mature peptide (Egan et al., 2003). Also, rs7124442 (T/T homozygotes) are associated with activity dependent BDNF secretion and BDNF mRNA trafficking (Egan et al., 2003; Orefice et al., 2013). In our previous work (Failla et al., 2015), a gene risk score (GRS) was used, ranking genetic variants associated with decreased BDNF production or secretion, as risk variants for mortality after TBI. These high risk genotypes were associated with increased mortality only among younger individuals with TBI (age <45 years). However, for the low-risk genotype there was an interaction by age, wherein younger individuals with low-risk variants (high secretion/trafficking) were protected against mortality, while older individuals with low-risk variants were at an increased risk for mortality after TBI.

The literature evaluating cortisol actions in acute TBI pathophysiology among clinical populations are limited. Our previous work demonstrates that serum cortisol levels are elevated over the first week after severe TBI, particularly among older individuals, as a physiological response to the acute stress of associated with severe injury and associated critical illness (Wagner et al., 2011a). Although, some develop acute adrenal insufficiency and a relative state of hypocortisolemia after injury (Wagner et al., 2011a). In contrast, our previous work also shows that acute CSF cortisol profiles are ~8–10X that observed among healthy controls over the first week after severe TBI. Further, persistent high acute CSF cortisol levels are associated both increased mortality as well as worse survivor based outcomes (Santarsieri et al., 2014). To date there are no clinical studies delineating possible regulatory effects cortisol may have on BDNF in the context of TBI.

Given the regulatory relationships between CORT and BDNF, and their known individual relationships to TBI outcome, we hypothesized that CSF cortisol would mediate CSF BDNF associations with 6-month mortality in a model that also considered age and BDNF genetics (e.g., as covariates or as points of cohort stratification). We also hypothesized that *BDNF* genetics would moderate the relationship between CSF BDNF and cortisol trajectories. Our results confirm these hypotheses and showed that CSF BDNF predicts, and BDNF GRS moderates, patient cortisol group-based trajectory analysis (TRAJ) group membership. Also, cortisol TRAJ predicted 6-month mortality. In mediation analysis, BDNF predicted mortality, with cortisol acting as the mediator, and CSF cortisol mediation effects were stronger among younger individuals (age <48 years) compared to older individuals. Together, the findings suggest a possible regulatory role for cortisol with BDNF's contribution to TBI pathophysiology and mortality, particularly among younger individuals with severe TBI.

## Materials and methods

### Participants

**Figure 1:**
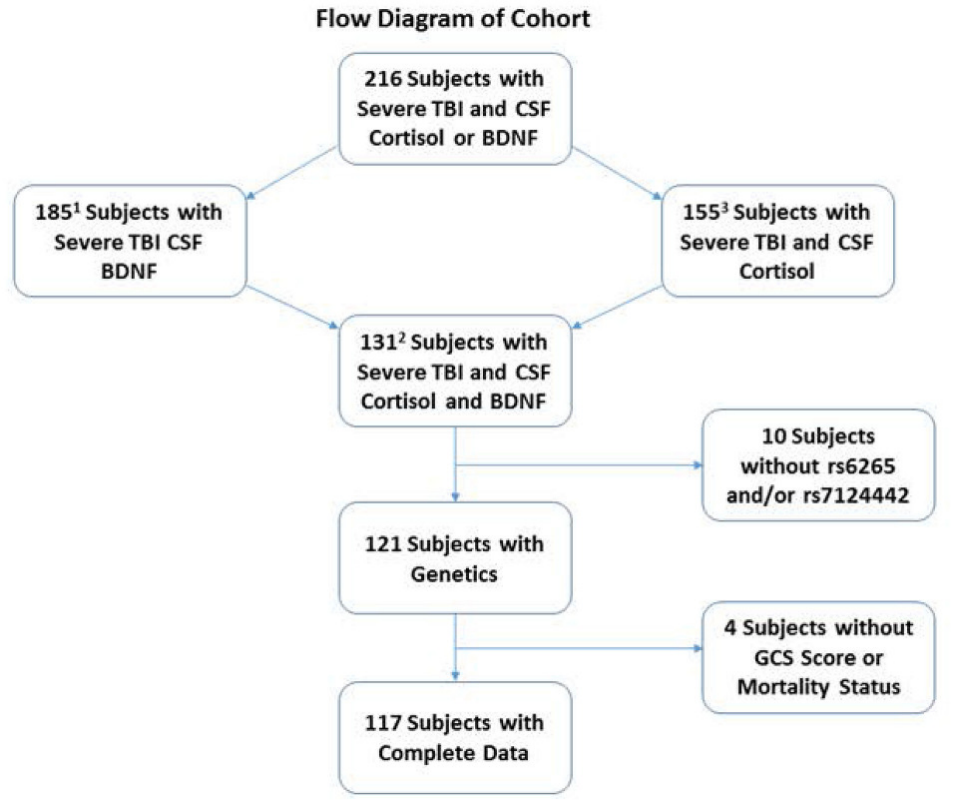
**Diagram depicting how the cohort was derived for analysis**. The cohort was restricted to self-reported White individuals with complete demographic, clinical, genetics and biomarker data. There were a total of 216 White severe TBI subjects with CSF Cortisol and BDNF. Of these, 185 and 155 subjects had CSF BDNF and cortisol, respectively. A total of 131 subjects had both CSF cortisol and BDNF. A total of 14 subjects were removed for missing BDNF genetics or GCS or mortality data, leaving a final cohort of 117. ^1^Used for estimating prior distributions in the mediation analysis path for BDNF-mortality (total effect). ^2^Used for estimating prior distributions in the mediation analysis path of BDNF-cortisol, cortisol-mortality, and BDNF-mortality (direct effect). ^3^Used to generate CSF cortisol trajectories in the most inclusive cohort with CSF cortisol.

This study was approved by the University of Pittsburgh Institutional Review Board, and this cohort represents a subset of patients enrolled in a larger study examining biomarker and genetic relationships to outcome after TBI. Participants were 16–73 years old and had a diagnosis of severe TBI [GCS≤8, with positive findings consistent with TBI on CT scan]. Self-reported non-White individuals were excluded as BDNF levels and genetics vary based on race (Freedman et al., 2004; Nettiksimmons et al., 2014). As shown in Figure 1, 216 participants had either BDNF *or* acute cortisol measurements; specifically, 185 had BDNF and 155 had cortisol. There were 131 participants with both BDNF and cortisol. Fourteen were excluded due to missing *BDNF* genotyping, GCS or mortality data. The final sample size was 117 participants with TBI. CSF from 13 healthy subjects was obtained and used to generate a control reference level for cortisol.

### CSF sample collection and processing

Brain-derived neurotropic factor (BDNF) and Cortisol were measured from CSF samples collected the first week post-injury. Samples were collected up to twice daily via EVD (extraventricular drainage) catheter placed for routine clinical care. For our injury cohort, 455 CSF samples were analyzed for BDNF, and 400 CSF samples were analyzed for cortisol. Healthy control subjects (*N* = 13) had CSF collected via lumbar puncture at ~7 a.m. After collection, samples were stored at 4°C until processing. Samples were then centrifuged, aliquoted, and stored at −80°C until batch analysis. CSF BDNF levels were measured via ELISA kit (RayBiotech), previously detailed (Failla et al., 2016). The inter/intra-assay coefficient of variation (CV) was <10% and <12%, respectively; assay sensitivity was 80 pg/mL.

CSF cortisol levels were measured in with solid-phase 125I RIA, Coat-A-Count® *in vitro* Diagnostic Test Kit (Siemens Healthcare Diagnostics). Inter- and intra-assay CVs were <10%. Samples with levels below the given range were assigned the detection limit value, and samples with undetectable levels were assigned values of 0.001 for analysis purposes. Additional samples were measured with a commercial ELISA kit (1-3002, Salimetrics) according to manufacturer instructions, with some adaptation for CSF measurement. To avoid matrix effects and to fit within the range of the kit, a 1:4 sample dilution was used. The inter-/intra-assay CV was <10% and <16%, respectively. Validation experiments with CSF also showed excellent linearity with serial dilution and recovery from spiking (90%–110%). Additionally, we measured cortisol levels by ELISA in 20 CSF samples previously measured by RIA to determine the correlation between the two measurement methods. A linear regression fit the relationship between the measured concentrations by RIA and ELISA. The ELISA results were then converted using this equation (*C*_*RIA*_ = 6.32 + 0.76 × *C*_*ELISA*_) before pooling data for analyses.

### Mortality

Six-month mortality status was examined using survival analysis. GOS scores were obtained by research-trained neuropsychometrists blinded to genetic and biomarker information. The Social Security Death Index (http://www.genealogybank.com/gbnk/ssdi/) was utilized to determine mortality status, and time post-injury until death.

### Genotyping and SNP selection

DNA was either isolated from blood using a simple salting out procedure or from CSF using the Qiamp DNA extraction protocol (Qiagen). Two *BDNF* SNPs were genotyped by TaqMan allele discrimination assay using commercial Assay-on-Demand reagents (Applied Biosystems Incorporated). Genotype data calls from two individuals, blinded to all phenotype data, were compared; discrepancies were settled by reviewing the raw data and rerunning samples when necessary. Hardy-Weinberg equilibrium was confirmed for all allele frequencies.

Brain derived neurotrophic factor *(BDNF)* rs6265 and rs7124442 were selected based on previous data correlating these SNPs to mortality. Both rs6265 and rs7124442 have been reported as functional with a minor allele frequency of 38.9 and 39.1%, respectively. Each SNP represents a different haplotype block of BDNF covering variation corresponding to BDNF isoform A (Failla et al., 2015). A risk allele count, GRS, was used as previously published (Failla et al., 2015) with the hypothesized risk alleles, for young individuals with TBI, being rs6265 Met and rs7124442 C carriers (Egan et al., 2003; Orefice et al., 2013). The GRS is fundamentally based upon the idea that genetic risk is cumulative. The cumulative GRS for the two identified *BDNF* variants ranged from 0 to 2 and was calculated by summing the number of risk alleles for these two SNPs. GRS = 0 was considered the no risk group (Val/Val, T/T), GRS = 1 contained carriers for one risk allele (Val/Val, C-carrier or T/T, Met-carrier), and GRS = 2 included carriers of both risk alleles (Met-carrier, C-carrier). This method for GRS formulation is consistent with GRIPS (Genetic RIsk Prediction Studies) guidelines (Janssens et al., 2011).

### Statistical analysis

Analysis was performed with SAS (Statistical Analysis Software) version 9.4 and the SPSS (Statistical Package for Social Sciences) version 23. Descriptive statistics included means, standard error of the mean (SEM), and frequencies. Mean comparisons for CSF biomarkers and continuous demographic and clinical variables were assessed using the Mann-Whitney *U*-test. Associations between categorical demographic and clinical variables by survival status were conducted using the Chi-square test, or Fisher's exact test (if applicable). The daily levels for CSF BDNF and cortisol were also graphically represented by survival status. Weekly average for BDNF were calculated by survival status and GRS.

### Trajectory analysis

To assess temporal (repeated measures) CSF cortisol profiles, we applied TRAJ (Niyonkuru et al., 2013) as previously reported (Santarsieri et al., 2014, 2015) to describe multiple longitudinal patterns of change in order to identify distinct subgroups within the population. For cortisol, TRAJ was conducted on natural log transformed values from 7 time points (D0–D6 post-TBI). Cortisol group TRAJ membership identified individuals with “*high*” vs. “*low*” cortisol level over this time frame.

For BDNF levels, we initially applied TRAJ procedures and found an unbalanced group membership (>85% participants belonged to the “*low*” group), indicating poor discrimination of participants into homogeneous subgroups for longitudinal BDNF levels. Also, upon inspecting daily levels, we observed stable BDNF concentrations over time; given the minimal temporal variation, we characterized mean BDNF above/below the 75th percentile for analysis.

### A bayesian approach to inform and internally validate mediation model

Attrition in sample size was a concern as the primary analysis was restricted to subjects with samples of BDNF, cortisol, BDNF genetics, GCS, and age. To protect against a selection bias of only using information from subjects with complete data, we used prior knowledge of the BDNF/mortality association to inform the current study analysis. Specifically, we applied the Bayes estimation using Monte Carlo Markov Chain (MCMC) method to assess primary relationships. The MCMC method, a stochastic procedure that estimates parameters of interest using random generated samples, can be used with Bayes estimation (Hamra et al., 2013). Using the MCMC method, we obtained exact parameter distributions of interest instead of asymptotic normal distribution requiring a medium or large sample size under maximum likelihood estimation. Final Bayes estimates rested between the prior distribution and the current effect size obtained from the study cohort for our mediation analysis. The MCMC procedure incorporates re-sampling of regions from prior distributions, to increase estimate precision and validity.

### Mediation and regression analyses

Mediation is used to test whether a factor is in the causal pathway between an exposure of interest and a selected outcome. We hypothesized that cortisol trajectory can mediate the relationship between BDNF and mortality. To formally test the hypothesis, we applied the Baron and Kenny method of mediation analysis (Baron and Kenny, 1986). In this method, a series of regression models were performed to adjust for other potential confounders.

To examine whether the association between acute CSF BDNF levels and mortality was mediated by acute CSF cortisol levels, we established if the following four criteria for mediation effects were met: (1) BDNF levels were associated with mortality (total effect); (2) BDNF levels were associated with cortisol levels; (3) cortisol levels were associated with mortality after adjusting for BDNF levels; and (4) the association between BDNF levels and mortality was attenuated after adjusting for cortisol level (indirect effect).

We performed logistic regression to evaluate the significance of criteria 2, and generated Cox proportional hazard models to evaluate the significance of criteria 1, 3, and 4. Time of follow-up was computed from injury and death date or 180 days (6-months) after injury, which represents the right censored time point. Regression and mediation analyses were performed for the entire population and after stratifying the population at the 75th percentile for age. Cox proportional hazard models were adjusted for binary 75th percentile age (<48 vs. ≥48 years old), GCS and GRS.

To investigate the moderating effects of GRS on BDNF level associations with cortisol TRAJ group, we performed a logistic regression model, stratified by GRS group (0 vs. 1 vs. 2). A two-way interaction term between BDNF level and GRS was created and fit into each model to assess the statistical significance of interactions.

The mediation percentage was calculated by considering the natural logarithm (ln) of the odds (or hazard) ratios (OR) with the following equation: Mediation percentage = {[ln(OR_TotalEffect_) − ln(OR_DirectEffect_)]/ln(OR _TotalEffect_)}^*^100%. Conceptually, the relationship between BDNF and mortality may be mediated by cortisol TRAJ, but this effect typically does not account for 100% of that relationship, and there may be other unmeasured factors that also mediate the relationship to some degree. The direct effect, corresponds to the effect of BDNF on mortality, adjusting for cortisol TRAJ. That is, if the mortality relationship through cortisol TRAJ is removed, the remaining effect represents BDNF effects on mortality without adjustment for cortisol TRAJ. The indirect effect, or the mediation effect, is derived by subtracting the total effect from the direct effect.

## Results

### Cohort demographics

There were 117 individuals with TBI in the final analytic sample (Figure 1). The clinical and demographics for the whole cohort, and stratified by survival is provided in Table 1. The age range for this cohort was 16–74, with the mean age 36 years old. Most subjects were men (85.3%). The median GCS was 7. The primary mechanisms of injury were automobile (52.2%) and motorcycle accidents (24.3%). The mean non-head Injury Severity Score was 13.26, while the average length of acute hospital stay was 21.2 days. The most prevalent neurological injury types were subarachnoid hemorrhage (SAH, 70.1%) and subdural hematoma (SDH, 69.2%). Individuals with TBI that were non-survivors were significantly older, had lower GCS scores (more severe injuries), fall mechanism of injuries, shorter length of stays, and were more likely to have contusions and less likely to have DAI in CT scans (*p* = 0.05 for all comparisons). No other demographic and clinical variables were significantly different by survival status.

**Table 1:** **Demographic and clinical characterization of the cohort**.

	**Overall (*n* = 117)**	**Survivors (*n* = 91)**	**Non-survivors (*n* = 26)**	***P*-value**
Age in years (mean ± SE)	36.0 ± 1.5	32.40 (1.4)	48.42 (3.7)	<0.001^*^
Sex, Men (%)	99 (85.3)	77 (85.6)	22 (84.6)	0.905
GCS, median (IQR)	7 (6-7)	7 (6-8)	6 (6-7)	0.043^*^
Mechanism of injury, *n* (%)				0.034^*^
Automobile	60 (52.2)	41 (45.6)	7 (26.9)	
Motorcycle	28 (24.3)	22 (24.4)	6 (23.1)	
Fall/jump	19 (16.5)	10 (11.1)	9 (34.6)	
Other	8 (7.0)	17 (18.9)	4 (15.4)	
Length of hospital stay in days (mean ± SE)	21.2 ± 1.0	23.01 (1.1)	14.68 (1.7)	0.002^*^
Injury Severity Score Non-head (mean ± SE)	13.26 (1.0)	12.99 (1.0)	14.18 (2.3)	0.822
Neurological injury type, *n* (%)				
SDH	81 (69.2)	60 (65.9)	21 (80.8)	0.148
DAI	36 (30.8)	33 (36.3)	3 (11.5)	0.016^*^
EDH	14 (12.0)	11 (12.1)	3 (11.5)	0.939
Contusion	52 (44.4)	35 (38.5)	17 (65.4)	0.015^*^
IVH	39 (33.3)	32 (35.2)	7 (30.0)	0.432
ICH	40 (34.2)	30 (33.0)	10 (38.5)	0.602
SAH	82 (70.1)	61 (67.0)	21 (80.8)	0.177
rs6265, *n* (%)				0.617
Val/Val	68 (58.1)	54 (59.3)	14 (53.9)	
Met-carrier	49 (41.9)	37 (40.7)	12 (46.2)	
rs7124442, *n* (%)				0.520
T/T	61 (52.1)	46 (50.6)	15 (57.7)	
C-carrier	56 (47.9)	45 (49.5)	11 (42.3)	
BDNF mean (SE)	0.19 (0.01)	0.18 (0.01)	0.21 (0.02)	0.045^*^
Cortisol mean (SE)	24.28 (1.3)	21.01 (1.20)	35.75 (3.45)	<0.0001^*^

* *p ≤ 0.05*.

### CSF cortisol trajectory profile

**Figure 2:**
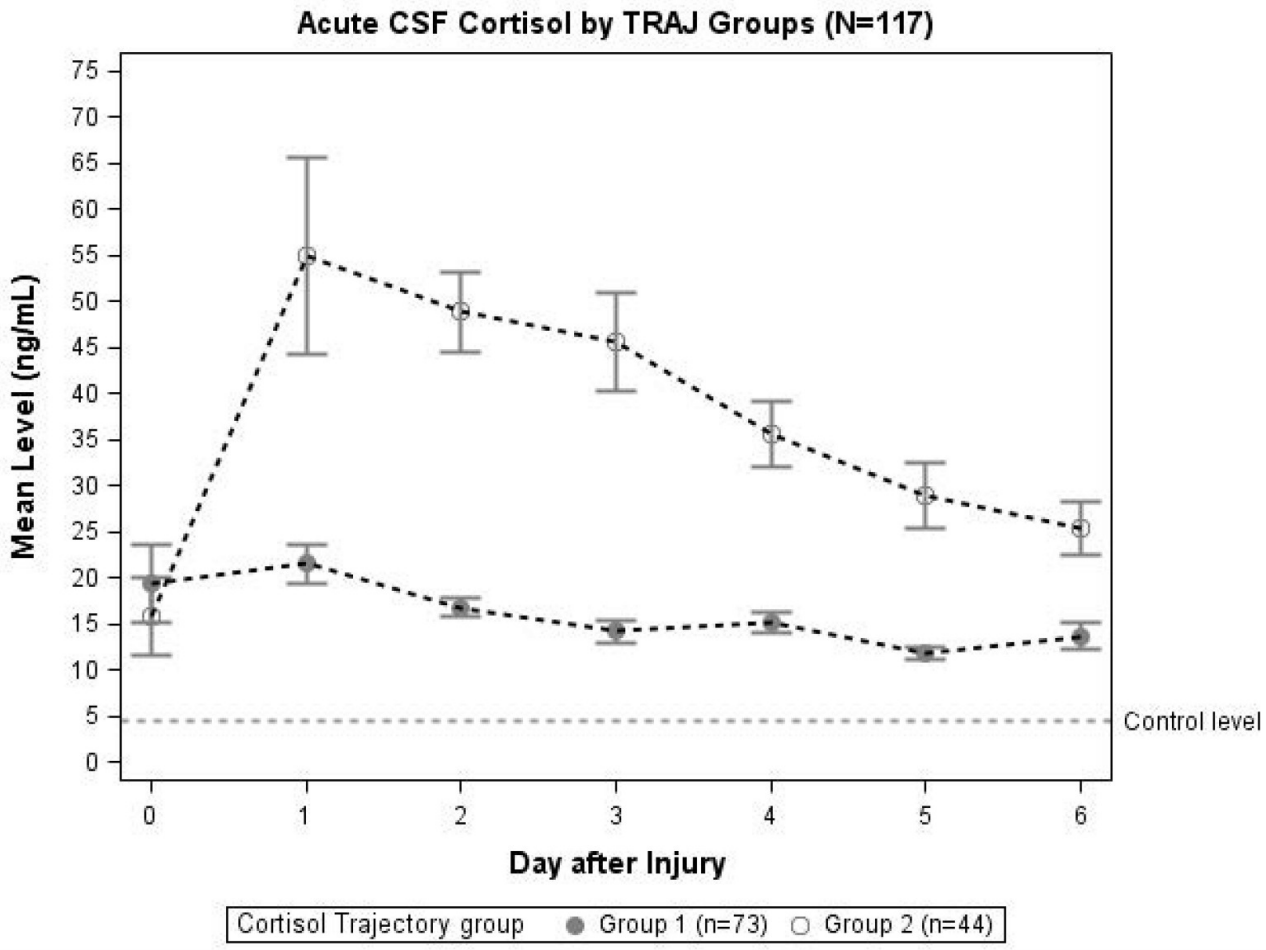
**Daily mean CSF cortisol levels for CSF cortisol TRAJ groups (*N* = 117)**. Daily mean CSF cortisol levels for the cortisol TRAJ groups. Error bars indicate standard error of the mean. There was a significant difference in CSF cortisol levels between the two groups on days 1–6 (*p* < 0.01). CSF cortisol values are provided for *N* = 13 healthy controls as a reference.

Trajectory analysis identified two different TRAJ group profiles: *high* and *low* CSF cortisol (Figure 2). CSF cortisol levels for the *high* group were higher than the *low* group on days 1–6 post-injury (*p* < 0.01 all comparisons). CSF cortisol levels in the *high* group peaked on day 2 post-injury and then declined, while levels for the *low* group were highest on day 1, followed by a modest decrease in levels. Also *high* CSF cortisol TRAJ group was associated with increased mortality (*P* = 0.001) when adjusting for age (<48 vs. ≥ 48 years old), GCS, and GRS. In the *high* cortisol TRAJ group, there were significant differences in CSF cortisol levels over time, with significant differences noted for day 0 vs. days 1–6 (*P* = 0.012 all comparisons). In the *low* cortisol TRAJ group, we observed a stably low level from day 0 to 6 with a small spike from day 0 to day 1 and a subsequent decline. There was no significant difference of average day 1–6 levels compared with day 0 (*P* = 0.219).

### Characterization of cortisol and BDNF profiles by survival status

**Figure 3:**
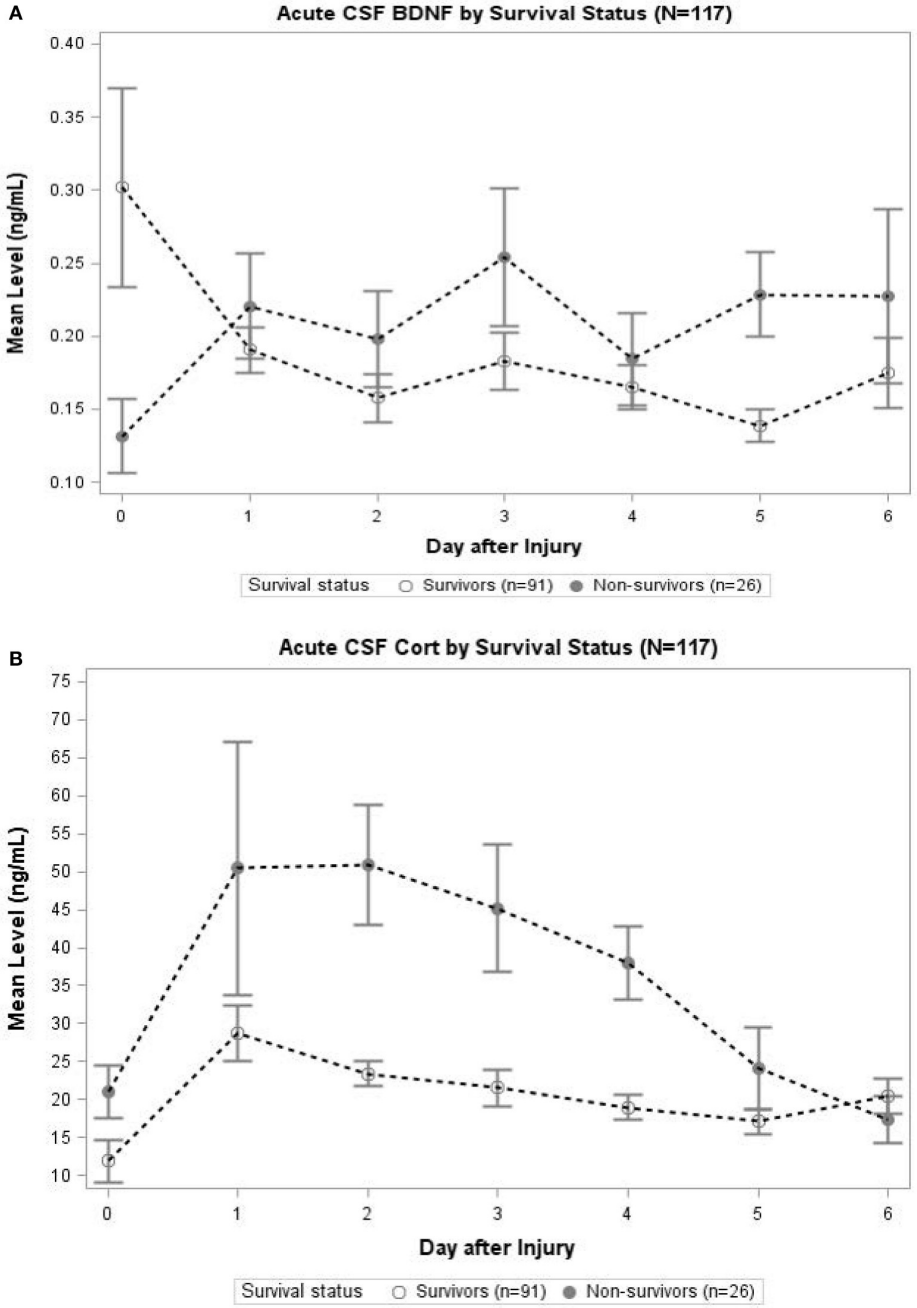
**(A)** Acute CSF Cortisol by Survival Status (*N* = 117). The open circles indicate survivors and closed circles are non-survivors. Data for CSF Cortisol (ng/mL) were averaged within survival category each day. Error bars indicate standard error of the mean. On days 2–4, CSF Cortisol levels were significantly (*p* < 0.05) elevated among non-survivors compared to survivors. **(B)** Acute CSF BDNF by Survival Status (*N* = 117). The open circles indicate survivors and closed circles are non-survivors. Data for CSF BDNF (ng/mL) were averaged within survival category each day. Error bars indicate standard error of the mean. On days 3 and 5, CSF BDNF levels were significantly (*p* < 0.05) elevated among non-survivors compared to survivors. There was a trend toward significant difference in levels on day 0 (*p* = 0.078); there were no significant differences in CSF BDNF levels for any other day.

In Figure 3A, daily cortisol levels were graphed by survival status. In days 2, 3, and 4, non-survivors had significantly elevated cortisol levels compared to survivors (*p* < 0.05 all comparisons). Daily levels of CSF BDNF were plotted by survival status (Figure 3B). Levels were significantly elevated among non-survivors compared to survivors at day 3 and 5 (*p* < 0.05). There was a trend toward lower BDNF levels for non-survivors compared to survivors at day 0 (*p* = 0.078). No other day was significantly different with respect to BDNF levels by survival status.

### Cortisol trajectory mediates BDNF associations with mortality

We obtained prior knowledge of the coefficient of each corresponding pathway in the mediation analysis from our original study cohort of all subjects with BDNF (*n* = 185). The summary of the prior knowledge used is provided in Table 2. Prior information showed those with a mean CSF BDNF level ≥75th percentile were more often in the *high* cortisol TRAJ group (*HR* = 4.12, 95% CI: 1.86, 9.24, *P* = 0.001), after adjusting for age, GCS and GRS. Mediation analysis, adjusting for binary age, GCS, and GRS, showed CSF BDNF levels dichotomized at the 75th percentile were associated with mortality (total effect) (*HR* = 1.94, 95% CI: 1.19, 3.12, *P* = 0.011). The CSF BDNF-mortality association was attenuated after adjusting for CSF cortisol TRAJ membership (direct effect) (*P* = 0.106), possibly suggesting a full mediation of CSF BDNF-mortality association through CSF cortisol. However, the proportion of mediation was ~30% (see Table 3); therefore, we report partial mediation effects of CSF cortisol. The mediation effect was stronger among younger individuals (≤48 years old) (mediation ~45%) when the population was stratified and evaluated in separate models (Table 3). There were no significant mediation effects among older individuals, although this group has a small sample size (*N* = 27).

**Table 2:** **Prior knowledge of BDNF and mortality association mediated by cortisol level among all subjects and stratified by 75th quartile of age**.

	**All subjects (*N* = 185)**		**Age ≤ 48 years (*N* = 139)**		**Age >48 years (*N* = 46)**	
**Pathway^a^**	**β (SE)**	***P*-value**	**β (SE)**	***P*-value**	**β (SE)**	***P*-value**
BDNF to Cortisol (Logistic)^b^	1.35 (0.48)	**0.005**	1.94 (0.61)	**0.001**	0.30 (0.75)	0.694
Cortisol to mortality (Cox)	1.13 (0.40)	**0.005**	1.00 (0.56)	0.095	1.27 (0.57)	**0.026**
BDNF to mortality-Total Effect (Cox)	0.65 (0.30)	**0.033**	1.03 (0.42)	**0.015**	0.30 (0.41)	0.457
BDNF to mortality-Direct Effect (Cox)^c^	0.47 (0.38)	0.217	0.54 (0.65)	0.407	0.48 (0.49)	0.324

a *All pathways adjusted for 75th percentile age (at 48 year old)*.

b *Weekly average CSF BDNF in ng/mL (≥75th percentile, <75th percentile); CSF cortisol using trajectory analysis grouping (low vs. high)*.

c *Also adjusted for cortisol trajectory grouping (low vs. high)*.

*Bolded p-values indicate statistical significance*.

**Table 3:** **Bayes estimation of BDNF and mortality association mediated by cortisol level among all subjects and by 75th percentile of age using Monte Carlo Markov Chain (MCMC) method^a^**.

	**All subjects (N = 117)**		**Age ≤ 48 years (*N* = 90)**		**Age >48 years (*N* = 27)**	
**Pathway^b^**	**HR/OR (95% CI)**	***P*-value**	**HR/OR (95% CI)**	***P*-value**	**HR/OR (95% CI)**	***P*-value**
BDNF to Cortisol (Logistic)	4.12 (1.86–9.24)	**0.001**	7.48 (3.22–17.92)	**<0.001**	1.44 (0.43–4.80)	0.185
Cortisol to mortality (Cox)	3.05 (1.70–5.45)	**<0.001**	2.99 (1.27–7.00)	**0.017**	3.61 (1.53–8.97)	**0.006**
BDNF to mortality-Total Effect (Cox)	1.94 (1.19–3.12)	**0.011**	2.89 (1.42–5.70)	**0.004**	1.47 (0.73–2.95)	0.221
BDNF to mortality-Direct Effect (Cox)	1.58 (0.90–2.78)	0.106	1.78 (0.72–4.42)	0.185	1.72 (0.78–3.76)	0.159
Mediation percentage (95% CI)^c^	29.92%		45.45%		−41.10%	
	(19.58–40.25%)		(34.05–56.85%)		(−93.52%, 11.33%)	

a *Analysis was performed with weekly average CSF BDNF in ng/mL (≥75th percentile, <75th percentile); cortisol using trajectory analysis grouping (low vs. high); Prior distribution of coefficients for each corresponding pathway (Table 2) was incorporated in MCMC Bayes estimation, which resamples prior distributions to derive a more precise and valid parameter estimate*.

b *All pathways were adjusted for 75th percentile age (<48 vs. ≥48 years old), Glasgow Coma Scale (GCS) and Gene Risk Score (GRS). For “BDNF to mortality-Direct Effect,” cortisol using trajectory analysis grouping (low vs. high) was additionally adjusted*.

c The mediation percentage was calculated with the following equation: Mediation percentage = {[ln(OR_Total Effect_) − ln(OR_Direct Effect_)]/ln(OR Total Effect)}^*^100.

*Bolded p-values indicate statistical significance*.

### BDNF biomarker associations with cortisol are dependent upon BDNF genotype

**Figure 4:**
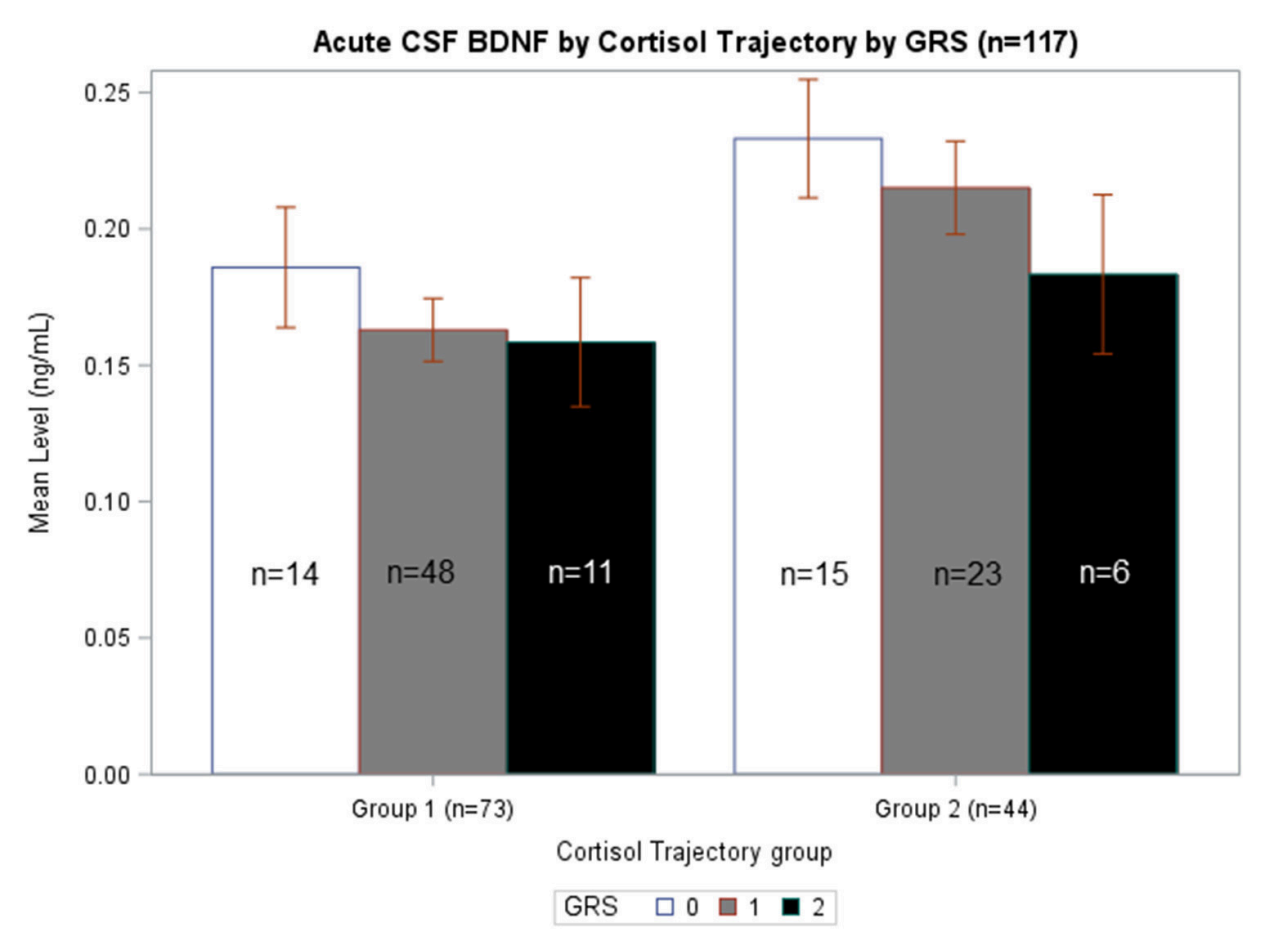
**Acute CSF BDNF by Cortisol Trajectory Group by GRS**. Weekly average CSF BDNF (ng/mL) is reported. Error bars indicate standard error of the mean. White, gray, and black corresponds to GRS scores of 0, 1, and 2, respectively. Results are stratified among the high (*n* = 44) and low (*n* = 73) cortisol TRAJ groups. Among individuals with GRS = 1, those in the high cortisol TRAJ group had significantly elevated BDNF levels compared to those in the low cortisol TRAJ group *p* = 0.011. There were no significant differences in BDNF levels by cortisol TRAJ group for individuals with GRS of 0 or 2.

We generated separate models among those with GRS = 0, GRS = 1, and GRS = 2 to evaluate the predictive capacity of CSF BDNF with regard to CSF cortisol TRAJ group membership. These age-adjusted models showed that, among subjects with GRS = 1, being in the high BDNF group (>75th percentile) was associated with *high* CSF cortisol TRAJ group membership (OR = 6.50, 95% CI: 1.70, 24.81, *P* = 0.006) (Table 4). In Figure 4, average BDNF levels were calculated by GRS in each cortisol TRAJ group. In GRS = 1 group, there is a significant difference in BDNF levels in CORT TRAJ high vs. low (*p* = 0.011). There is no statistically significant difference for GRS 0 or 2. These findings are consistent with the results from Table 4, wherein the GRS = 2 group was relatively small in sample size.

**Table 4:** **Odds Ratios and 95% Confidence Intervals for CSF BDNF and cortisol levels**.

	**GRS = 0 (*N* = 29)^a^**		**GRS = 1 (*N* = 71)**		**GRS = 2 (*N* = 17)**	
	**Cortisol TRAJ Group 2 vs. 1^b^**	**OR (95% CI)^c^**	**Cortisol TRAJ Group 2 vs. 1^b^**	**OR (95% CI)**	**Cortisol TRAJ Group 2 vs. 1^b^**	**OR (95% CI)**
BDNF 75th pct.	15/14	2.46 (0.30, 16.13)	23/48	6.50 (1.70, 24.81)	6/11	9.00 (0.28, 285.51)
*P* trend		0.285		**0.006**		0.213

*Individual Models stratified by Gene Risk Scores*.

a *GRS score: 0 = Val/Val (rs6265) and T/T (rs7124442); 1 = Val/Val (rs6265), C-carriers (rs7124442) or Met-Carriers (rs6265), T/T (rs7124442); 2 = Met-Carriers (rs6265) and C-Carriers (rs7124442)*.

b *Number of subjects in each cortisol trajectory group (Group 2 has higher cortisol level)*.

c *Adjusted for binary age (≤48 vs. >48 years old)*.

*Bolded p-value indicates statistical significance*.

### BDNF genetic interactions with CSF BDNF level mortality relationships may be age dependent

An age stratified analysis, with separate models for age <48 vs. ≥48 years old, revealed younger individuals had a significant CSF BDNF^*^GRS interaction in this mortality model (Table 5, *P* = 0.028). This model was adjusted for GCS and cortisol TRAJ group. For younger people, this interaction shows the association between high BDNF and mortality varies by *BDNF* genotype such that those with higher secretion genotypes (GRS = 0) have a higher mortality risk.

**Table 5:** **Cox proportional hazards model for the association between BDNF and mortality^a^**.

	**Age below 75th percentile (≤48 years) (*N* = 90)**		**Age above 75th percentile (>48 years) (*N* = 27)**	
	**Coefficient (SE)**	***P*-value**	**Coefficient (SE)**	***P*-value**
BDNF^*^GRS	−2.28 (1.04)	**0.028**	−0.26 (1.13)	0.819

*Individual models are stratified by age*.

a *Covariates included in the model: GCS, cortisol (trajectory group)*.

*Bolded p-value indicates statistical significance*.

**Figure 5:**
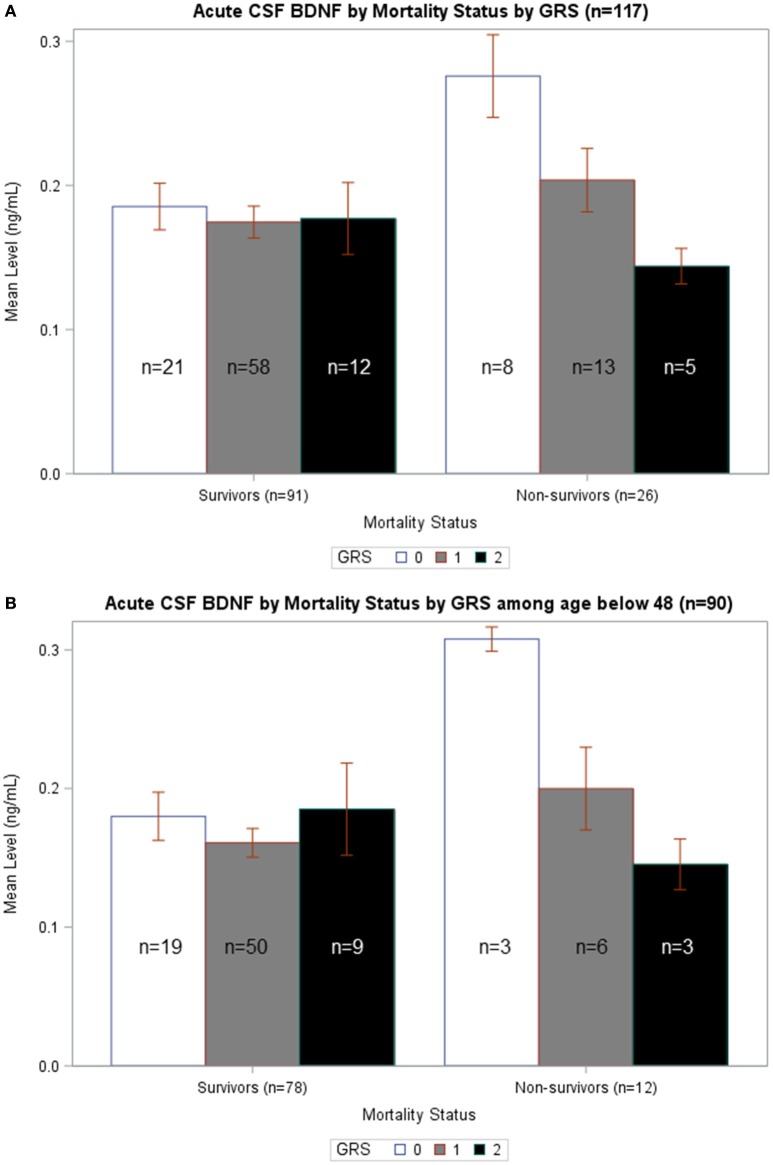
**(A)** Acute CSF BDNF by Mortality Status by GRS. Weekly average CSF BDNF (ng/mL) is reported. Error bars indicate standard error of the mean. White, gray, and black corresponds to GRS scores of 0, 1, and 2, respectively. Results are stratified among survivors (*n* = 91) and non-survivors (*n* = 26). Among non-survivors, there is a significant difference in BDNF levels by GRS, such that higher GRS scores have lowest BDNF levels (*p* = 0.015). There is no difference in BDNF levels by GRS in survivors-only. Only among individuals with GRS scores of 0 is there a significant difference in BDNF levels in survivors compared to non-survivors (*p* = 0.007). **(B)** Acute CSF BDNF by Mortality Status by GRS among age below 48. Weekly average CSF BDNF (ng/mL) in reported after restricting population to only individuals below age 48. Error bars indicate standard error of the mean. White, gray, and black corresponds to GRS scores of 0, 1, and 2, respectively. Results are stratified among survivors (*n* = 78) and non-survivors (*n* = 12). Among non-survivors, there is a significant difference in BDNF levels by GRS, such that higher GRS scores have lowest BDNF levels (*p* = 0.032). There is no difference in BDNF levels by GRS in survivors-only. Only among individuals with GRS scores of 0 is there a significant difference in BDNF levels in survivors compared to non-survivors (*p* = 0.015).

In Figure 5A, average BDNF levels were calculated by survival status and GRS. In non-survivors, there is a significant difference in mean BDNF levels by GRS (*p* = 0.015), such that individuals with GRS of 2 had the lowest BDNF levels and individuals with GRS of 0 had the highest BDNF. Also, non-survivors with a GRS of 0 had significantly increased BDNF compared to survivors with a GRS of 0 (*p* = 0.007). In Figure 5B, BDNF levels by survival status and GRS were calculated among just individuals <48 years old. In non-survivors, there was a significant difference in BDNF levels by GRS, with individuals with GRS of 2 having the lowest BDNF (*p* = 0.032). Among individuals with GRS of 0, BDNF levels were significantly elevated among non-survivors compared to survivors (*p* = 0.015). There were no significant differences in BDNF levels by GRS among either survivors or non-survivors over the age of 48 (data not shown). Together, these data are consistent with the multivariate model in Table 5 and suggest CSF cortisol has a regulatory influence on younger individuals with high BDNF levels and a low BDNF secretion genotype.

## Discussion

Cortisol and BDNF are crucial biomarkers to characterize in the context of TBI, as they can have both potentially useful and harmful effects in the brain. This study investigated the relationship between CSF cortisol and BDNF as gene- and age-dependent biomarkers that predict mortality after TBI. We found cortisol mediates BDNF effects on mortality after TBI and that genetics and age also influence this mediation effect. While our previous work shows cortisol and BDNF are predictive biomarkers for outcome post-TBI (Santarsieri et al., 2014; Failla et al., 2016), no studies have investigated the inter-relationships between these biomarkers, and their combined effects on clinical outcome. These data show the direct effects of high BDNF levels on mortality are more pronounced among younger individuals with TBI who also have high secretion *BDNF* genotypes. However, the regulatory (mediating) effects of high CSF cortisol on BDNF mortality risk occurs among those with low BDNF secretion genotypes.

We also found increased cortisol levels are associated with mortality after TBI. Elevated CSF cortisol is linked to an increased inflammatory response among adults with severe TBI (Santarsieri et al., 2015). While traditionally considered as having anti-inflammatory properties (Barnes, 1979), recent work suggests under certain conditions, CNS cortisol creates a more permissive, pro-inflammatory state (Sapolsky et al., 2000; Yeager et al., 2004). In addition to inflammation, cortisol has signaling and regulatory influences on neurotrophins (McEwan, 2012), particularly BDNF (Rothman and Mattson, 2013).

A prolonged, elevated cortisol state, which occurs with ongoing stress and is further perpetuated by impaired negative feedback secondary to the stress response, can result in reduced synaptic plasticity, mediated by glucocorticoid downregulation of BDNF expression (Duman et al., 1997; Numakawa et al., 2009). The focus on BDNF effects on learning and memory has garnered significant attention for BDNF due to its role in plasticity and neural maintenance, but BDNF is a viable biomarker for mortality as first reported by Failla et al. (2015).

Many studies have shown an inverse relationship between cortisol and BDNF in the setting of chronic stress and in experiments with glucocorticoid injections, where elevated cortisol leads to BDNF reductions (Smith et al., 1995) and adrenalectomy results in elevated BDNF levels (Stranahan et al., 2011). Our current study found positive associations between elevated CSF cortisol and BDNF levels. A closer, temporal examination of the stress literature demonstrates that acute stress increases BDNF (Marmigère et al., 2003; Tapia-Arancibia et al., 2004), while chronic stress reduces BDNF (Smith et al., 1995; Liston and Gan, 2011). This acute increase in BDNF has been confirmed in experimental TBI models across multiple brain regions (Yang et al., 1996; Hicks et al., 1997; Grundy et al., 2000; Rostami et al., 2014), while regional reductions in BDNF have also been documented at chronic time points after experimental TBI (Chen et al., 2005).

This initial increase in BDNF may represent a physiological response to influence HPA reactivity and attempt to maintain homeostasis within the brain in response to initial/acute stress (Marmigère et al., 2003; McEwan, 2015). However, BDNF increases can be pathological in the setting of high and/or prolonged pathophysiological stress (e.g., TBI). Clinically, increased CSF BDNF may be due to BBB disruption after TBI. In addition to brain production, BDNF is synthesized in the periphery (Caporali and Emanueli, 2009) and can be stored and released from platelets (Nakahashi et al., 2000) after tissue injury (Fujimura et al., 2002). Failla et al. (2015) demonstrated that serum BDNF levels are reduced post-TBI, hypothesizing that reduced serum levels may be due BDNF transit into the CNS, resulting in an acute increase in CSF BDNF. In either case, increased CSF BDNF may represent failed compensatory mechanisms that result in higher risk for mortality.

Acutely elevated CSF BDNF levels may not represent neuroprotective processes due to injury induced regional changes in TrkB vs. p75 target receptor expression. BDNF is released as proBDNF, which binds to the pro-apoptotic p75^NTR^. When cleaved by plasmin producing mature BDNF, this molecule binds to the pro-survival TrkB receptor. Experimental excitotoxic conditions, characteristic of TBI, have demonstrated a downregulation of full-length tyrosine kinase receptor (TrkB.FL) and an upregulation of the inactive truncated form of the receptor (TrkB.T) (Gomes et al., 2012; Vidaurre et al., 2012). A regionally specific increase in p75^NTR^ receptors additionally contributes to a shift from pro-survival to pro-apoptotic BDNF function, possibly contributing to increased mortality after injury. Aging also can detrimentally shift the BDNF receptor ratios, along with causing regional reductions in BDNF and TrkB mRNA expression (Romanczyk et al., 2002; Webster et al., 2006), including in the hypothalamus (Tapia-Arancibia et al., 2004). This age-related decline in BDNF pro-survival signaling may possibly hinder neuronal survival and maintenance, therefore increasing mortality risk. Age, and possibly also injury, related increases in p75^NTR^ receptors may contribute to less apparent cortisol mediation effects on BDNF associations with mortality.

Cortisol mediation of BDNF effects on mortality may be more prominent among those with high BDNF levels and concurrent low BDNF secretion genotypes, and the reported mediation effect suggest that there may be a mechanism connecting these two signaling pathways in the context of TBI mortality. One plausible mechanism might involve post-TBI cortisol elevations inhibiting the interaction of Shp2 with TrkB, affecting neurological injury severity. Cortisol mediated inhibition of this signaling pathway suppresses the MAPK/ERK pathway (Kumamaru et al., 2011), essential for transcribing certain neural plasticity genes (Arango-Lievano et al., 2015). Disrupted TrkB signaling could increase brain tissue damage even while elevated glucocorticoid levels are simultaneously increasing pro-BDNF and tissue plasminogen activator expression (Revest et al., 2014), to generate mature BDNF.

Another possible mechanism for cortisol mediated BDNF mortality effects is through autonomic instability after injury. TBI results in a marked disruption in autonomic balance and stability. Evidence of reduced HRV after TBI implies reduced parasympathetic tone (Baguley et al., 2006). With TBI, HRV has been shown to predict brain death and cerebrovascular dysregulation (Ryan et al., 2011), and HRV is associated with autonomic dysfunction in multiple other pathological states including sepsis, shock, and adrenal insufficiency (Morris et al., 2007; Werdan et al., 2009), each of which can co-occur in TBI. Further work suggests that HRV can be an effective mortality predictor, among hemodynamically stable patients with TBI (Ryan et al., 2011). Importantly, there is evidence that BDNF levels (Pal et al., 2014), *BDNF* genetic variability (Yang et al., 2010), and cortisol levels (Pal et al., 2014) are associated with HRV, making autonomic instability a plausible mechanism by which cortisol regulation of BDNF affects mortality risk.

Our previous work defined “risk alleles” as the genetic variations of the *BDNF* SNPs rs6265 and rs7124442 that result in lower activity-dependent BDNF secretion and impaired BDNF mRNA trafficking, respectively (Egan et al., 2003; Orefice et al., 2013), to create a GRS (Failla et al., 2015). Interestingly, higher GRSs were only associated with BDNF related mortality among younger subjects. A more nuanced look at the relationship between cortisol and BDNF in our population showed very high BDNF levels are associated with high cortisol levels among people who have a low secretion “risk allele” (rs6265 Met or rs7124442 C). Among these individuals, high CSF BDNF levels were associated with *high* CSF CORT TRAJ group membership. Our previous work suggests these risk variants are associated with lower serum BDNF. Previous work elaborated that, in addition to lower BDNF secretion capacity and autonomic mediated suppression of serum BDNF, low serum BDNF post-TBI may be due to reactive platelet BDNF dumping systemically and more BBB BDNF transit into the CNS (Tanno et al., 1992; Failla et al., 2016). Taken together, cortisol may have a regulatory influence (i.e., mediation effect) under conditions where innate BDNF production capacity is reduced, yet CNS BDNF levels are high due to injury severity factors, such as cortisol induced suppression of Shp2 associated MAPK/ERK signaling, reactive platelet dumping of BDNF systemically, and increased serum-to-CSF BBB transit. Our work is consistent with previous studies where those with rs6265 Met/Met homozygosity possessed significantly higher HPA axis reactivity, determined by serum cortisol elevations in response to physical stress (Schüle et al., 2006).

Our study demonstrates age contributes to biomarker associations on outcome after TBI. Age can affect many different biomarkers, including stress hormones (Wagner et al., 2011a; Santarsieri et al., 2014; Ranganathan et al., 2016), sex hormones (Ranganathan et al., 2016), inflammation (Kumar et al., 2016), and BDNF (Failla et al., 2015). Experimental models show decreased BDNF mRNA, BDNF protein, and TrkB.FL mRNA in many different brain regions with older age (Romanczyk et al., 2002; Webster et al., 2006; Erickson et al., 2012). Our study demonstrated that in younger subjects (age <48 year), CSF BDNF effects on mortality can differ as a function of genetic variation, while this BDNF^*^GRS interaction is not significant among older subjects. Lower sample size for the older subgroup, and for the GRS = 2 subgroup, are potential alternative reasons for this finding. While specific mechanisms are difficult to discern with these clinical biomarker data, age related differences in BDNF secretion capacity (perhaps due to age-dependent risk factors like hypertension, cerebral hypoperfusion, and poor glucose metabolism) (Kennedy et al., 2009; Erickson et al., 2012), age related increases in CSF BDNF acutely (Failla et al., 2015), and also target receptor milieu (Erickson et al., 2012) may contribute to this finding. What the data do suggest though is that, in the context of TBI, factors other than genetics contribute to CSF BDNF profiles observed in this age group.

While BDNF secretion capacity is reduced with aging, CSF BDNF levels are elevated after TBI and are increased with age (Failla et al., 2015). Although CSF cortisol relationships to CSF BDNF were studied, both BDNF and cortisol are synthesized in the periphery and likely contribute to the CSF profiles observed. Notably, BDNF levels are reduced in serum after TBI and are associated with mortality (Failla et al., 2015), while serum cortisol profiles are not associated with mortality (Wagner et al., 2011a). Thus, it is unclear at this point, how serum relationships between BDNF and cortisol might differ from CSF relationships between these two markers and why. Presumably, variable levels of BBB dysfunction, acute adrenal insufficiency associated with the critical illness that accompanies severe TBI, and personal biology may influence these relationships, creating the possibility for potentially heterogeneous or dynamic relationships with the role that CSF cortisol plays in the causal pathway between CSF BDNF and mortality.

The study has several limitations, including small sample size, particularly for age stratified analyses and for the BDNF cut-point. Dividing the age groups at the 75th percentile left the model underpowered to fully explore GRS^*^CSF BDNF interactions in the older age group, and larger future studies focusing on this older population would be beneficial. We used a data driven approach to determine the 75th percentile as the cut point for BDNF analyses. Specifically, the 75th percentile cutoff was based on our preliminary analysis where we used a larger, and presumably more representative cohort, to define the cut-point to be used with the Bayesian model. We believe the 75th percentile is a good starting estimate for examining high BDNF levels in other populations. It is possible that the actual cut point may vary in other populations (70th, 80th, percentile, etc.). However, our main interpretation of this work is that cortisol levels mediate associations between high BDNF levels and mortality.

Due to effects of race on BDNF allelic frequencies, we limited our analyses to self-reported Whites. Also, the group with a GRS = 2 is small, making significant associations with cortisol TRAJ challenging despite the large odds ratio. Despite this, some issues with low sample size are overcome by using the MCMC method of Bayes estimation, which uses prior knowledge to inform a posterior probability based on the current study data; it also incorporates a validation process to internally re-sample prior distributions to arrive at more precise and valid parameters. Future studies could consider a weighted GRS score formulation to better quantify the relative contribution each variant contributes to genetic associations identified in this study. Additionally, our small sample size limited our ability to differentiate *BDNF* heterozygote gene effects from variant homozygote gene effects on mortality. Examining *BDNF* heterozygote gene effects, including unique interactions with cortisol, may be relevant to TBI pathophysiology (Kim et al., 2011; Notaras et al., 2016).

Including only self-reported whites is an additional limitation as the results may not be generalizable to other races. However, the literature is clear that racial genetic differences can confound the relationships assessed (Freedman et al., 2004; Nettiksimmons et al., 2014). Only two *BDNF* gene variants were used to construct the GRS, which likely underestimates the total variance explained by *BDNF* genetics on cortisol profiles, BDNF profiles, and mortality; nevertheless, the chosen variants are both functional and affect BDNF (Egan et al., 2003; Orefice et al., 2013) and cortisol (Alexander et al., 2010; Armbruster et al., 2016) levels clinically. Additionally, our BDNF ELISA does not differentiate between the proBDNF and mature BDNF isoforms.

**Figure 6:**
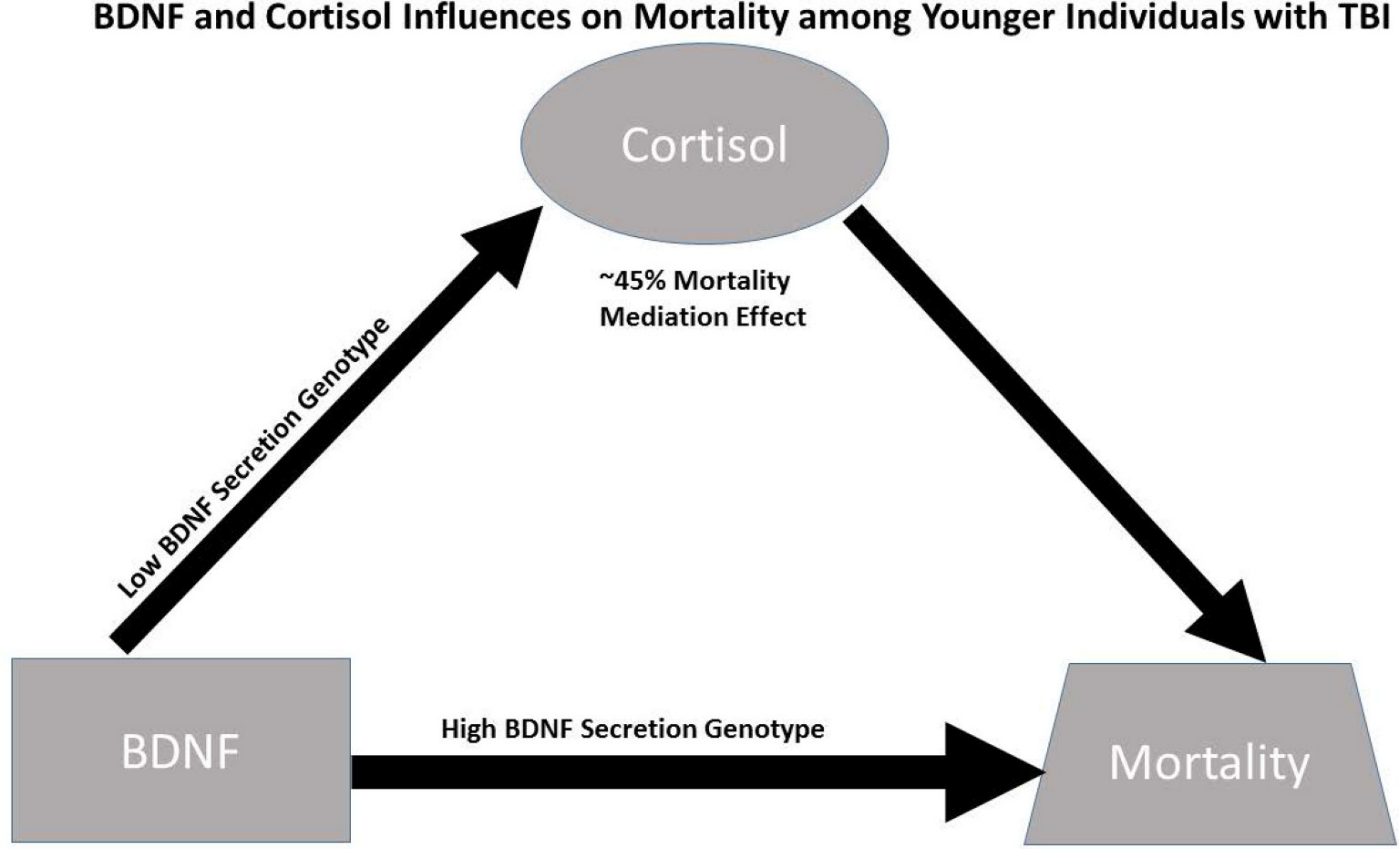
**Conceptual figure capturing biomarker relationships among younger individuals (≤48 years. Old, *N* = 90):** (1) cortisol has a significant regulatory (mediating) effect on BDNF's influence on mortality, accounting for ~45% of BDNF's mortality effects; (2) the association between BDNF above/below the 75th percentile and high cortisol TRAJ is only significant among individuals with low BDNF secretion genotypes; and (3) the direct association between BDNF above/below the 75th percentile and mortality risk is stronger among individuals with the high BDNF secretion genotype (GRS = 0) compared to the low secretion genotypes. Taken together, the data support a conceptual model for younger individuals with TBI wherein high BDNF levels among those with high BDNF secretion phenotypes directly increases mortality risk, while mortality risk for those with high BDNF levels paired with low BDNF secretion genotypes is regulated (mediated) by cortisol.

Despite these limitations, this study suggests causal effects of cortisol on mortality for those with high BDNF that are more pronounced among younger individuals with low BDNF secretion genotypes. While this causal relationship is important for mortality prediction post-TBI, specific mechanisms facilitating this relationship have yet to be discerned; additionally the high BDNF secretion genotype directly increases mortality risk among younger individuals with high BDNF levels (see Figure 6). In addition to injury effects on both CSF BDNF and cortisol biomarker levels (e.g., BBB damage), mechanisms by which these biomarkers interact with each other, age, and genetics to impact mortality are likely varied, but may reflect both neurological injury severity and autonomic instability. Future experimentation should evaluate how our mediation effects translate mechanistically to brain tissue BDNF and BDNF target receptor signaling after TBI. Additional trauma to body regions outside the head is common in the population with severe TBI. These injuries, as well as other systemic complications, such as hospital acquired pneumonia, sepsis, and shock can be non-neurological contributors to mortality. Thus, future work considering if/how serum BDNF or Cortisol reflect the peripheral injury response, and its effects on TBI pathophysiology is warranted. Clinically, it would be interesting to further characterize BDNF cortisol relationships, with survivor based outcomes, like learning and memory, from the viewpoint of post-acute TBI serving as a chronic stress paradigm influencing regulatory relationships between cortisol and BDNF. Understanding these dynamic relationships of secondary injury occurring after TBI may facilitate more effective, personalized interventions after injury.

## Ethics statement

All subjects gave written informed consent in accordance with the Declaration of Helsinki. The protocol was approved by the University of Pittsburgh Institutional Review Board.

## Author contributions

AW: Contributed to hypothesis formulation, literature review, results interpretation, writing and editing of manuscript. MM: Contributed to literature review, results interpretation, data generation, writing and editing of manuscript. MF: Contributed to hypothesis formulation, literature review, data generations, editing of manuscript. RK: Contributed to data analysis, results interpretation, writing and editing of manuscript. ZW: Contributed to data analysis, results interpretation, writing and editing of manuscript. YC: Contributed to data generation, results interpretation, and editing of manuscript. BO: Contributed to data generation, results interpretation and editing of manuscript.

## Funding

This work was supported in part by the Department of Defense (DOD W81XWH-071-0701); National Institutes of Health (NIH R01 HD048162, R01NR008424, 5P01NS030318; Centers for Disease Control (CDC) R49 CCR 323155); National Institute for Disability, Independent Living, and Rehabilitation Research (NIDILRR 90DP0041).

### Conflict of interest statement

The authors declare that the research was conducted in the absence of any commercial or financial relationships that could be construed as a potential conflict of interest.

## Abbreviations

BBB: blood brain barrier
BDNF: brain-derived neurotropic factor
CREB: cAMP response element binding protein
ELISA: enzyme-linked immunosorbent assay
GCS: Glasgow Coma Scale
GOS: Glasgow Outcome Scale
GRS: gene risk score
HPA axis: hypothamic-pituitary-adrenal axis
HRV: heart rate variability
MAPK/ERK: microtubule associated protein kinase/extracellular signal-regulated kinases
NT3: Neurotrophin-3
RCS: restricted cubic spline analysis
RIA: radioimmunoassay
Shp2: Src Homology Phosphatase 2
SNP: single nucleotide polymorphism
TBI: traumatic brain injury
TRAJ: group-based trajectory analysis
TrkB: tropomyosin receptor kinase B.

## References

[B1] Alboni S. Tascedda F. Corsini D. Benatti C. Caggia F. Capone G.. (2011). Stress induces altered CRE/CREB pathway activity and BDNF expression in the hippocampus of glucocorticoid receptor-impaired mice. Neuropharmacology 60, 1337–1346. 10.1016/j.neuropharm.2011.01.050 21324325

[B2] Alexander N. Osinsky R. Schmitz A. Mueller E. Kuepper Y. Hennig J. (2010). The BDNF Val66Met polymorphism affects HPA-axis reactivity to acute stress. Psychoneuroendocrinology 35, 949–953. 10.1016/j.psyneuen.2009.12.008 20079575

[B3] Antonawich F. J. Miller G. Rigsby D. C. Davis J. N. (1999). Regulation of ischemic cell death by glucocorticoids and adrenocorticotropic hormone. Neuroscience 88, 319–325. 10.1016/S0306-4522(98)00213-9 10051210

[B4] Arango-Lievano M. Lambert W. M. Bath K. G. Garabedian M. J. Chao M. V. Jeanneteau F. (2015). Neurotrophic-priming of glucocorticoid receptor signaling is essential for neuronal plasticity to stress and antidepressant treatment. Proc. Natl. Acad. Sci. U.S.A. 112, 15737–15742. 10.1073/pnas.1509045112 26630005 PMC4697403

[B5] Armbruster D. Müller-Alcazar A. Strobel A. Lesch K.-P. Kirschbaum C. Brocke B. (2016). BDNF val^66^met genotype shows distinct associations with the acoustic startle reflex and the cortisol stress response in young adults and children. Psychoneuroendocrinology 66, 39–46. 10.1016/j.psyneuen.2015.12.020 26773399

[B6] Baguley I. J. Heriseanu R. E. Felmingham K. L. Cameron I. D. (2006). Dysautonomia and heart rate variability following severe traumatic brain injury. Brain Inj. 20, 437–444. 10.1080/02699050600664715 16716989

[B7] Barnes P. J. (1979). Anti-inflammatory actions of glucocorticoids: molecular mechanisms. Clin. Sci. Lond. Engl. 94, 557–572. 9854452 10.1042/cs0940557

[B8] Baron R. M. Kenny D. A. (1986). The moderator-mediator variable distinction in social psychological research: conceptual, strategic, and statistical considerations. J. Pers. Soc. Psychol. 51, 1173–1182. 10.1037/0022-3514.51.6.1173 3806354

[B9] Blaha G. R. Raghupathi R. Saatman K. E. McIntosh T. K. (2000). Brain-derived neurotrophic factor administration after traumatic brain injury in the rat does not protect against behavioral or histological deficits. Neuroscience 99, 483–493. 10.1016/S0306-4522(00)00214-1 11029540

[B10] Caporali A. Emanueli C. (2009). Cardiovascular actions of neurotrophins. Physiol. Rev. 89, 279–308. 10.1152/physrev.00007.2008 19126759 PMC2836529

[B11] Centers for Disease Control Prevention (2016). Traumatic Brain Injury in the United States: Emergency Department Visits, Hospitalizations and Deaths 2002–2006. Available online at: http://www.cdc.gov/traumaticbraininjury/tbi_ed.html (Accessed April 6, 2016).

[B12] Chen X. Li Y. Kline A. E. Dixon C. E. Zafonte R. D. Wagner A. K. (2005). Gender and environmental effects on regional brain-derived neurotrophic factor expression after experimental traumatic brain injury. Neuroscience 135, 11–17. 10.1016/j.neuroscience.2005.05.041 16084663

[B13] Clark C. G. Hasser E. M. Kunze D. L. Katz D. M. Kline D. D. (2011). Endogenous brain-derived neurotrophic factor in the nucleus tractus solitarius tonically regulates synaptic and autonomic function. J Neurosci. 31, 12318–12329. 10.1523/JNEUROSCI.0746-11.2011 21865474 PMC3408222

[B14] Duman R. S. Heninger G. R. Nestler E. J. (1997). A molecular and cellular theory of depression. Arch. Gen. Psychiatry 54, 597–606. 10.1001/archpsyc.1997.01830190015002 9236543

[B15] Egan M. F. Kojima M. Callicott J. H. Goldberg T. E. Kolachana B. S. Bertolino A.. (2003). The BDNF val66met polymorphism affects activity-dependent secretion of BDNF and human memory and hippocampal function. Cell 112, 257–269. 10.1016/S0092-8674(03)00035-7 12553913

[B16] Erickson K. I. Miller D. L. Roecklein K. A. (2012). The aging hippocampus: interactions between exercise, depression, and BDNF. Neuroscientist 18, 82–97. 10.1177/1073858410397054 21531985 PMC3575139

[B17] Failla M. D. Conley Y. P. Wagner A. K. (2016). Brain-Derived Neurotrophic Factor (BDNF) in traumatic brain injury-related mortality: interrelationships between genetics and acute systemic and central nervous system BDNF profiles. Neurorehabil. Neural Repair. 30, 83–93. 10.1177/1545968315586465 25979196 PMC4644728

[B18] Failla M. D. Kumar R. G. Peitzman A. B. Conley Y. P. Ferrell R. E. Wagner A. K. (2015). Variation in the BDNF gene interacts with age to predict mortality in a prospective, longitudinal cohort with severe TBI. Neurorehabil. Neural Repair. 29, 234–246. 10.1177/1545968314542617 25063686 PMC4305354

[B19] Ferrari E. Cravello L. Muzzoni B. Casarotti D. Paltro M. Solerte S. B.. (2001). Age-related changes of the hypothalamic-pituitary-adrenal axis: pathophysiological correlates. Eur. J. Endocrinol. 144, 319–329. 10.1530/eje.0.1440319 11275940

[B20] Freedman M. L. Reich D. Penney K. L. McDonald G. J. Mignault A. A. Patterson N.. (2004). Assessing the impact of population stratification on genetic association studies. Nat. Genet. 36, 388–393. 10.1038/ng1333 15052270

[B21] Fujimura H. Altar C. A. Chen R. Nakamura T. Nakahasi T. Kambayashi J.. (2002). Brain-derived neurotrophic factor is stored in human platelets and released by agonist stimulation. Thromb. Haemost. 87, 728–734. 12008958

[B22] Gomes J. R. Costa J. T. Melo C. V. Felizzi F. Monteiro P. Pinto M. J.. (2012). Excitotoxicity downregulates TrkB.FL signaling and upregulates the neuroprotective truncated TrkB receptors in cultured hippocampal and striatal neurons. J. Neurosci. 32, 4610–4622. 10.1523/JNEUROSCI.0374-12.2012 22457507 PMC6622054

[B23] Goyal A. Failla M. D. Niyonkuru C. Amin K. Fabio A. Berger R. P.. (2013). S100b as a prognostic biomarker in outcome prediction for patients with severe traumatic brain injury. J Neurotrauma. 30, 946–957. 10.1089/neu.2012.2579 23190274 PMC3684103

[B24] Grundy P. L. Patel N. Harbuz M. S. Lightman S. L. Sharples P. M. (2000). Glucocorticoids modulate BDNF mRNA expression in the rat hippocampus after traumatic brain injury. Neuroreport 11, 3381–3384. 10.1097/00001756-200010200-00023 11059906

[B25] Grundy P. L. Patel N. Harbuz M. S. Lightman S. L. Sharples P. M. (2001). Glucocorticoids modulate the NGF mRNA response in the rat hippocampus after traumatic brain injury. Brain Res. 892, 386–390. 10.1016/S0006-8993(00)03258-3 11172788

[B26] Hamra G. MacLehose R. Richardson D. (2013). Markov chain Monte Carlo: an introduction for epidemiologists. Int. J. Epidemiol. 42, 627–634. 10.1093/ije/dyt043 23569196 PMC3619958

[B27] Hicks R. R. Numan S. Dhillon H. S. Prasad M. R. Seroogy K. B. (1997). Alterations in BDNF and NT-3 mRNAs in rat hippocampus after experimental brain trauma. Brain Res. Mol. Brain. Res. 48, 401–406. 10.1016/S0169-328X(97)00158-7 9332737

[B28] Janssens A. C. J. W. Ioannidis J. P. A. van Duijn C. M. Little J. Khoury M. J. GRIP Group (2011). Strengthening the reporting of genetic risk prediction studies: The GRIPS Statement. Ann. Intern. Med. 154, 421–425. 10.7326/0003-4819-154-6-201103150-00008 21403077

[B29] Jeanneteau F. D. Lambert W. M. Ismaili N. Bath K. G. Lee F. S. Garabedian M. J.. (2012). BDNF and glucocorticoids regulate corticotrophin-releasing hormone (CRH) homeostasis in the hypothalamus. Proc. Natl. Acad. Sci. U.S.A. 109, 1305–1310. 10.1073/pnas.1114122109 22232675 PMC3268297

[B30] Jeanneteau F. Garabedian M. J. Chao M. V. (2008). Activation of Trk neurotrophin receptors by glucocorticoids provides a neuroprotective effect. Proc. Natl. Acad. Sci. U.S.A. 105, 4862–4867. 10.1073/pnas.0709102105 18347336 PMC2290769

[B31] Joëls M. (2008). Functional actions of corticosteroids in the hippocampus. Eur. J. Pharmacol. 583, 312–321. 10.1016/j.ejphar.2007.11.064 18275953

[B32] Kennedy K. M. Rodrigue K. M. Land S. J. Raz N. (2009). BDNF Val66Met polymorphism influences age differences in microstructure of the Corpus Callosum. Front. Hum. Neurosci. 3:19. 10.3389/neuro.09.019.2009 19738930 PMC2737488

[B33] Kim J.-M. Stewart R. Bae K.-Y. Kim S.-W. Yang S.-J. Park K.-H.. (2011). Role of BDNF val66met polymorphism on the association between physical activity and incident dementia. Neurobiol. Aging 32, 551.e5–e12. 10.1016/j.neurobiolaging.2010.01.018 20172629

[B34] Kumamaru E. Numakawa T. Adachi N. Kunugi H. (2011). Glucocorticoid suppresses BDNF-stimulated MAPK/ERK pathway via inhibiting interaction of Shp2 with TrkB. FEBS Lett. 585, 3224–3228. 10.1016/j.febslet.2011.09.010 21946312

[B35] Kumamaru E. Numakawa T. Adachi N. Yagasaki Y. Izumi A. Niyaz M.. (2008). Glucocorticoid prevents brain-derived neurotrophic factor-mediated maturation of synaptic function in developing hippocampal neurons through reduction in the activity of mitogen-activated protein kinase. Mol. Endocrinol. 22, 546–558. 10.1210/me.2007-0264 18096693 PMC5419617

[B36] Kumar R. G. Diamond M. L. Boles J. A. Berger R. P. Tisherman S. A. Kochanek P. M.. (2015). Acute CSF interleukin-6 trajectories after TBI: associations with neuroinflammation, polytrauma, and outcome. Brain Behav. Immun. 45, 253–262. 10.1016/j.bbi.2014.12.021 25555531

[B37] Kumar R. G. Rubin J. E. Berger R. P. Kochanek P. M. Wagner A. K. (2016). Principal components derived from CSF inflammatory profiles predict outcome in survivors after severe traumatic brain injury. Brain Behav. Immun. 53, 183–193. 10.1016/j.bbi.2015.12.008 26705843 PMC4783208

[B38] Liston C. Gan W.-B. (2011). Glucocorticoids are critical regulators of dendritic spine development and plasticity *in vivo*. Proc. Natl. Acad. Sci. U.S.A. 108, 16074–16079. 10.1073/pnas.1110444108 21911374 PMC3179117

[B39] Maas A. I. R. Roozenbeek B. Manley G. T. (2010). Clinical trials in traumatic brain injury: past experience and current developments. Neurother. J. Am. Soc. Exp. Neurother. 7, 115–126. 10.1016/j.nurt.2009.10.022 20129503 PMC5084118

[B40] Marmigère F. Givalois L. Rage F. Arancibia S. Tapia-Arancibia L. (2003). Rapid induction of BDNF expression in the hippocampus during immobilization stress challenge in adult rats. Hippocampus 13, 646–655. 10.1002/hipo.10109 12921353

[B41] McEwan B. S. (1999). Stress and the aging hippocampus. Front. Neuroendocrinol. 20, 49–70. 10.1006/frne.1998.0173 9882536

[B42] McEwan B. S. (2012). The ever-changing brain: cellular and molecular mechanisms for the effects of stressful experiences. Dev. Neurobiol. 72, 878–890. 10.1002/dneu.20968 21898852 PMC3248634

[B43] McEwan B. S. (2015). Preserving neuroplasticity: role of glucocorticoids and neurotrophins via phosphorylation. Proc. Natl. Acad. Sci. U.S.A. 112, 15544–15545. 10.1073/pnas.1521416112 26627713 PMC4697404

[B44] Morris J. A. Norris P. R. Waitman L. R. Ozdas A. Guillamondegui O. D. Jenkins J. M. (2007). Adrenal insufficiency, heart rate variability, and complex biologic systems: a study of 1,871 critically ill trauma patients. J. Am. Coll. Surg. 204, 885–892. 10.1016/j.jamcollsurg.2007.01.019 17481504

[B45] Nakahashi T. Fujimura H. Altar C. A. Li J. Kambayashi J. Tandon N. N.. (2000). Vascular endothelial cells synthesize and secrete brain-derived neurotrophic factor. FEBS Lett. 470, 113–117. 10.1016/S0014-5793(00)01302-8 10734218

[B46] Nettiksimmons J. Simonsick E. M. Harris T. McDonald G. J. Andre Mignault A. A. Patterson N.. (2014). The associations between serum brain-derived neurotrophic factor, potential confounders, and cognitive decline: a longitudinal study. PLoS ONE 9:e91339. 10.1371/journal.pone.0091339 24670553 PMC3966768

[B47] Niyonkuru C. Wagner A. K. Ozawa H. Amin K. Goyal A. Fabio A. (2013). Group-based trajectory analysis applications for prognostic biomarker model development in severe TBI: a practical example. J. Neurotrauma. 30, 938–945. 10.1089/neu.2012.2578 23421760

[B48] Notaras M. J. Hill R. A. Gogos J. A. van den Buuse M. (2016). BDNF Val66Met genotype interacts with a history of simulated stress exposure to regulate sensorimotor gating and startle reactivity. Schizophr. Bull. 10.1093/schbul/sbw077 27262112 PMC5464110

[B49] Numakawa T. Kumamaru E. Adachi N. Yagasaki Y. Izumi A. Kunugi H. (2009). Glucocorticoid receptor interaction with TrkB promotes BDNF-triggered PLC-gamma signaling for glutamate release via a glutamate transporter. Proc. Natl. Acad. Sci. U.S.A. 106, 647–652. 10.1073/pnas.0800888106 19126684 PMC2626757

[B50] Orefice L. L. Waterhouse E. G. Partridge J. G. Lalchandani R. R. Vicini S. Xu B. (2013). Distinct roles for somatically and dendritically synthesized brain-derived neurotrophic factor in morphogenesis of dendritic spines. J. Neurosci. 33, 11618–11632. 10.1523/JNEUROSCI.0012-13.2013 23843530 PMC3724547

[B51] Pal R. Singh S. N. Chatterjee A. Saha M. (2014). Age-related changes in cardiovascular system, autonomic functions, and levels of BDNF of healthy active males: role of yogic practice. Age (Dordr) 36:9683. 10.1007/s11357-014-9683-7 25012275 PMC4150910

[B52] Ranganathan P. Kumar R. G. Davis K. McCullough E. H. Berga S. L. Wagner A. K. (2016). Longitudinal sex and stress hormone profiles among reproductive age and post-menopausal women after severe TBI: a case series analysis. Brain Inj. 30, 452–461. 10.3109/02699052.2016.1144081 26963638

[B53] Revest J.-M. Le Roux A. Roullot-Lacarrière V. Kaouane N. Vallee M. Kasanetz F.. (2014). BDNF-TrkB signaling through Erk1/2 MAPK phosphorylation mediates the enhancement of fear memory induced by glucocorticoids. Mol. Psychiatry. 19, 1001–1009. 10.1038/mp.2013.134 24126929 PMC4195976

[B54] Roberts I. Yates D. Sandercock P. Farrell B. Wasserberg J. Lomas G.. (2004). Effect of intravenous corticosteroids on death within 14 days in 10008 adults with clinically significant head injury (MRC CRASH trial): randomised placebo-controlled trial. Lancet Lond Engl. 364, 1321–1328. 10.1016/S0140-6736(04)17188-2 15474134

[B55] Romanczyk T. B. Weickert C. S. Webster M. J. Herman M. M. Akil M. Kleinman J. E. (2002). Alterations in trkB mRNA in the human prefrontal cortex throughout the lifespan. Eur. J. Neurosci. 15, 269–280. 10.1046/j.0953-816x.2001.01858.x 11849294

[B56] Roozenbeek B. Chiu Y.-L. Lingsma H. F. Gerber L. M. Steyerberg E. W. Ghajar J.. (2012). Predicting 14-day mortality after severe traumatic brain injury: application of the IMPACT models in the brain trauma foundation TBI-trac® New York State database. J Neurotrauma. 29, 1306–1312. 10.1089/neu.2011.1988 22150207 PMC3335134

[B57] Rostami E. Krueger F. Plantman S. Davidsson J. Agoston D. Grafman J.. (2014). Alteration in BDNF and its receptors, full-length and truncated TrkB and p75(NTR) following penetrating traumatic brain injury. Brain Res. 1542, 195–205. 10.1016/j.brainres.2013.10.047 24192075

[B58] Rothman S. M. Mattson M. P. (2013). Activity-dependent, stress-responsive BDNF signaling and the quest for optimal brain health and resilience throughout the lifespan. Neuroscience 239, 228–240. 10.1016/j.neuroscience.2012.10.014 23079624 PMC3629379

[B59] Ryan M. L. Ogilvie M. P. Pereira B. M. T. Gomez-Rodriguez J. C. Manning R. J. Vargas P. A.. (2011). Heart rate variability is an independent predictor of morbidity and mortality in hemodynamically stable trauma patients. J. Trauma. 70, 1371–1380. 10.1097/TA.0b013e31821858e6 21817974

[B60] Santarsieri M. Kumar R. G. Kochanek P. M. Berga S. Wagner A. K. (2015). Variable neuroendocrine-immune dysfunction in individuals with unfavorable outcome after severe traumatic brain injury. Brain Behav Immun. 45, 15–27. 10.1016/j.bbi.2014.09.003 25218898 PMC4342288

[B61] Santarsieri M. Niyonkuru C. McCullough E. H. Dobos J. A. Dixon E. C. Berga S. L.. (2014). Cerebrospinal fluid cortisol and progesterone profiles and outcomes prognostication after severe traumatic brain injury. J. Neurotrauma. 31, 699–712. 10.1089/neu.2013.3177 24354775 PMC3967414

[B62] Sapolsky R. M. Romero L. M. Munck A. U. (2000). How do glucocorticoids influence stress responses? Integrating permissive, suppressive, stimulatory, and preparative actions. Endocr. Rev. 21, 55–89. 10.1210/edrv.21.1.0389 10696570

[B63] Schüle C. Zill P. Baghai T. C. Eser D. Zwanzger P. Wenig N.. (2006). Brain-derived neurotrophic factor Val66Met polymorphism and dexamethasone/CRH test results in depressed patients. Psychoneuroendocrinology 31, 1019–1025. 10.1016/j.psyneuen.2006.06.002 16890377

[B64] Sebastiani A. Gölz C. Werner C. Schäfer M. K. E. Engelhard K. Thal S. C. (2015). Proneurotrophin binding to P75 neurotrophin receptor (P75ntr) is essential for brain lesion formation and functional impairment after experimental traumatic brain injury. J Neurotrauma. 32, 1599–1607. 10.1089/neu.2014.3751 25879397

[B65] Smith M. A. Makino S. Kvetnansky R. Post R. M. (1995). Stress and glucocorticoids affect the expression of brain-derived neurotrophic factor and neurotrophin-3 mRNAs in the hippocampus. J. Neurosci. 15(3 Pt 1), 1768–1777. 7891134 10.1523/JNEUROSCI.15-03-01768.1995 PMC6578156

[B66] Stranahan A. M. Arumugam T. V. Mattson M. P. (2011). Lowering corticosterone levels reinstates hippocampal brain-derived neurotropic factor and Trkb expression without influencing deficits in hypothalamic brain-derived neurotropic factor expression in leptin receptor-deficient mice. Neuroendocrinology 93, 58–64. 10.1159/000322808 21160171 PMC3066242

[B67] Tanno H. Nockels R. P. Pitts L. H. Noble L. J. (1992). Breakdown of the blood-brain barrier after fluid percussive brain injury in the rat. Part 1: Distribution and time course of protein extravasation. J. Neurotrauma. 9, 21–32. 10.1089/neu.1992.9.21 1619673

[B68] Tapia-Arancibia L. Aliaga E. Silhol M. Arancibia S. (2008). New insights into brain BDNF function in normal aging and Alzheimer disease. Brain Res. Rev. 59, 201–220. 10.1016/j.brainresrev.2008.07.007 18708092

[B69] Tapia-Arancibia L. Rage F. Givalois L. Arancibia S. (2004). Physiology of BDNF: focus on hypothalamic function. Front. Neuroendocrinol. 25, 77–107. 10.1016/j.yfrne.2004.04.001 15571756

[B70] Vidaurre O. G. Gascón S. Deogracias R. Sobrado M. Cuadrado E. Montaner J.. (2012). Imbalance of neurotrophin receptor isoforms TrkB-FL/TrkB-T1 induces neuronal death in excitotoxicity. Cell Death Dis. 3:e256. 10.1038/cddis.2011.143 22258407 PMC3270277

[B71] Wagner A. K. Amin K. B. Niyonkuru C. Postal B. A. McCullough E. H. Ozawa H.. (2011b). CSF Bcl-2 and cytochrome C temporal profiles in outcome prediction for adults with severe TBI. J. Cereb. Blood Flow Metab. 31, 1886–1896. 10.1038/jcbfm.2011.31 21448217 PMC3185877

[B72] Wagner A. K. Fabio A. Puccio A. M. Hirschberg R. Li W. Zafonte R. D.. (2005). Gender associations with cerebrospinal fluid glutamate and lactate/pyruvate levels after severe traumatic brain injury. Crit. Care Med. 33, 407–413. 10.1097/01.CCM.0000153931.23488.DD 15699846

[B73] Wagner A. K. McCullough E. H. Niyonkuru C. Ozawa H. Loucks T. L. Dobos J. A.. (2011a). Acute serum hormone levels: characterization and prognosis after severe traumatic brain injury. J Neurotrauma. 28, 871–888. 10.1089/neu.2010.1586 21488721 PMC3113446

[B74] Wagner A. K. Ren D. Conley Y. P. Ma X. Kerr M. E. Zafonte R. D.. (2007). Sex and genetic associations with cerebrospinal fluid dopamine and metabolite production after severe traumatic brain injury. J. Neurosurg. 106, 538–547. 10.3171/jns.2007.106.4.538 17432702

[B75] Webster M. J. Herman M. M. Kleinman J. E. Shannon Weickert C. (2006). BDNF and trkB mRNA expression in the hippocampus and temporal cortex during the human lifespan. Gene Expr. Patterns 6, 941–951. 10.1016/j.modgep.2006.03.009 16713371

[B76] Werdan K. Schmidt H. Ebelt H. Zorn-Pauly K. Koidl B. Hoke R. S.. (2009). Impaired regulation of cardiac function in sepsis, SIRS, and MODS. Can. J. Physiol. Pharmacol. 87, 266–274. 10.1139/Y09-012 19370080

[B77] Yang A. C. Chen T.-J. Tsai S.-J. Hong C. J. Kuo C. H. Yang C. H.. (2010). BDNF Val66Met polymorphism alters sympathovagal balance in healthy subjects. Am. J. Med. Genet. B Neuropsychiatr. Genet. 153B, 1024–1030. 10.1002/ajmg.b.31069 20213725

[B78] Yang K. Perez-Polo J. R. Mu X. S. Yan H. Q. Xue J. J. Iwamoto Y. (1996). Increased expression of brain-derived neurotrophic factor but not neurotrophin-3 mRNA in rat brain after cortical impact injury. J Neurosci Res. 44, 157–164. 10.1002/(SICI)1097-4547(19960415)44:2<157::AID-JNR8>3.0.CO;2-C 8723224

[B79] Yeager M. P. Guyre P. M. Munck A. U. (2004). Glucocorticoid regulation of the inflammatory response to injury. Acta Anaesthesiol. Scand. 48, 799–813. 10.1111/j.1399-6576.2004.00434.x 15242423

